# Supplementary material for: Allosteric fine-tuning of the conformational equilibrium poises the chaperone BiP for post-translational regulation
Source: eLife. 2017 Oct 24;6:e29430. doi: 10.7554/eLife.29430 (PMC5655141; doi:10.7554/eLife.29430)
Supplement: Figure 4—source data 1. — The percentage of the population of the domain-docked conformation was calculated from methyl peak intensities of three non-overlapping peak doublets (P1, P2 and P3), each containing peaks for the domain-docked (D) and -undocked (U) conformations, using the following equation: pD=IDID+IU×100%, where ID and IU are the intensities of peaks corresponding to the domain-docked and -undocked conformations, respectively. Errors were set as standard deviations (SDs) from the means for three doublets or uncertainties from the errors in peak intensities, whatever is larger. BiPT229G (ATP-bound): pD = 53 ± 6.6%. [file elife-29430-fig4-data1.docx]

**Figure 4–source data 1**

**NMR analysis of populations for the domain-docked and -undocked conformations.**

The percentage of populations of the domain-docked conformation were calculated from methyl peak intensities of three non-overlapping peak doublets (P1, P2 and P3), each containing peaks for the domain-docked (D) and -undocked (U) conformations, using the following equation: $p_{D}=\frac{I_{D}}{I_{D}+I_{U}}\times100\%$, where I_D_ and I_U_ are the intensities of peaks corresponding to the domain-docked and -undocked conformations, respectively. Errors were set as standard deviations (SDs) from the means for three doublets or uncertainties from the errors in peak intensities, whatever is larger.

**BiPT229G (ATP-bound):** p_D_=53±6.6%

| P1 | | P2 | | P3 | |
| --- | --- | --- | --- | --- | --- |
| I_U_ | I_D_ | I_U_ | I_D_ | I_U_ | I_D_ |
| 1.54E+06  ±8.21E+04 | 2.50E+06  ±8.21E+04 | 1.73E+06  ±8.21E+04 | 1.47E+06  ±8.21E+04 | 1.74E+06  ±8.21E+04 | 1.79E+06  ±8.21E+04 |

**BiPT229G (ATP-and P2-bound):** p_D_=30±8.0%

| P1 | | P2 | | P3 | |
| --- | --- | --- | --- | --- | --- |
| I_U_ | I_D_ | I_U_ | I_D_ | I_U_ | I_D_ |
| 2.24E+06  ±8.52E+04 | 1.54E+06  ±8.52E+04 | 2.41E+06  ±8.52E+04 | 6.66E+05  ±8.52E+04 | 2.48E+06  ±8.52E+04 | 9.49E+05  ±8.52E+04 |

**BiPT229G (ADP-bound):** p_D_=19±7.1%

| P1 | | P2 | | P3 | |
| --- | --- | --- | --- | --- | --- |
| I_U_ | I_D_ | I_U_ | I_D_ | I_U_ | I_D_ |
| 2.92E+06  ±8.02E+04 | 6.13E+05  ±8.02E+04 | 3.87E+06  ±8.02E+04 | 4.80E+05  ±8.02E+04 | 2.26E+06  ±8.02E+04 | 8.91E+05  ±8.02E+04 |

**BiPT229GV461F (ATP-bound):** p_D_=66±4.8%

| P1 | | P2 | | P3 | |
| --- | --- | --- | --- | --- | --- |
| I_U_ | I_D_ | I_U_ | I_D_ | I_U_ | I_D_ |
| 1.79E+06  ±5.62E+04 | 4.63E+06  ±5.62E+04 | 2.15E+06  ±5.62E+04 | 3.29E+06  ±5.62E+04 | 1.83E+06  ±5.62E+04 | 3.57E+06  ±5.62E+04 |

**BiPT229GV461F (ADP-bound):** p_D_=49±10.7%

| P1 | | P2 | | P3 | |
| --- | --- | --- | --- | --- | --- |
| I_U_ | I_D_ | I_U_ | I_D_ | I_U_ | I_D_ |
| 2.18E+06  ±5.73+04 | 3.01E+06  ±5.73+04 | 4.04E+06  ±5.73+04 | 2.04E+06  ±5.73+04 | 2.03E+06  ±5.73+04 | 2.39E+06  ±5.73+04 |

**BiPT229GI526V (ATP-bound):** p_D_=61±5.0%

| P1 | | P2 | | P3 | |
| --- | --- | --- | --- | --- | --- |
| I_U_ | I_D_ | I_U_ | I_D_ | I_U_ | I_D_ |
| 1.09E+06  ±9.95E+04 | 2.07E+06  ±9.95E+04 | 1.08E+06  ±9.95E+04 | 1.26E+06  ±9.95E+04 | 9.78E+05  ±9.95E+04 | 1.67E+06  ±9.95E+04 |

**BiPT229GI526V (ADP-bound):** p_D_=35±8.1%

| P1 | | P2 | | P3 | |
| --- | --- | --- | --- | --- | --- |
| I_U_ | I_D_ | I_U_ | I_D_ | I_U_ | I_D_ |
| 9.27E+05  ±9.77E+04 | 5.35E+05  ±9.77E+04 | 1.23E+06  ±9.77E+04 | 3.96E+05  ±9.77E+04 | 7.32E+05  ±9.77E+04 | 5.75E+05  ±9.77E+04 |

**BiPT229GI437V (ATP-bound):** p_D_=29±3.4%

| P1 | | P2 | | P3 | |
| --- | --- | --- | --- | --- | --- |
| I_U_ | I_D_ | I_U_ | I_D_ | I_U_ | I_D_ |
| 1.84E+06  ±9.79E+04 | 9.48E+05  ±9.79E+04 | 1.96E+06  ±9.79E+04 | 7.76E+05  ±9.79E+04 | 2.25E+06  ±9.79E+04 | 7.86E+05  ±9.79E+04 |

**BiPT229GI437V (ADP-bound):** p_D_=N/A*

*No noticeable peaks were observed for the domain-docked conformation, suggesting that its population is less than ~10%.

**BiPT229GI538V (ATP-bound):** p_D_=34±4.5%

| P1 | | P2 | | P3 | |
| --- | --- | --- | --- | --- | --- |
| I_U_ | I_D_ | I_U_ | I_D_ | I_U_ | I_D_ |
| 2.07E+06  ±9.86E+04 | 1.29E+06  ±9.86E+04 | 2.03E+06  ±9.86E+04 | 7.91E+05  ±9.86E+04 | 1.93E+06  ±9.86E+04 | 1.10E+06  ±9.86E+04 |

**BiPT229GI538V (ADP-bound):** p_D_=N/A*

*No noticeable peaks were observed for the domain-docked conformation, suggesting that its population is less than ~10%.
