# Supplementary material for: Allosteric fine-tuning of the conformational equilibrium poises the chaperone BiP for post-translational regulation
Source: eLife. 2017 Oct 24;6:e29430. doi: 10.7554/eLife.29430 (PMC5655141; doi:10.7554/eLife.29430)
Supplement: Figure 5—source data 1. — The percentage of populations of the domain-docked conformation were calculated from methyl peak intensities of three non-overlapping peak doublets (P1, P2 and P3), each containing peaks for the domain-docked (D) and -undocked (U) conformations, using the following equation: pD=IDID+IU×100%, where ID and IU are the intensities of peaks corresponding to the domain-docked and -undocked conformations, respectively. Errors were set as standard deviations (SDs) from the means for three doublets or uncertainties from the errors in peak intensities, whatever is larger. [file elife-29430-fig5-data1.docx]

**Figure 5–source data 1**

**NMR analysis of populations for the domain-docked and -undocked conformations for AMPylated BiP.**

The percentage of populations of the domain-docked conformation were calculated from methyl peak intensities of three non-overlapping peak doublets (P1, P2 and P3), each containing peaks for the domain-docked (D) and -undocked (U) conformations, using the following equation: $p_{D}=\frac{I_{D}}{I_{D}+I_{U}}\times100\%$, where I_D_ and I_U_ are the intensities of peaks corresponding to the domain-docked and -undocked conformations, respectively. Errors were set as standard deviations (SDs) from the means for three doublets or uncertainties from the errors in peak intensities, whatever is larger.

**BiPT229G AMPylated (ATP-bound):** p_D_=68±3.9%

| P1 | | P2 | | P3 | |
| --- | --- | --- | --- | --- | --- |
| I_U_ | I_D_ | I_U_ | I_D_ | I_U_ | I_D_ |
| 2.38E+06  ±7.13E+04 | 6.23E+06  ±7.13E+04 | 2.95E+06  ±7.13E+04 | 5.00E+06  ±7.13E+04 | 2.48E+06  ±7.13E+04 | 5.36E+06  ±7.13E+04 |

**BiPT229G AMPylated (ADP-bound):** p_D_=64±7.9%

| P1 | | P2 | | P3 | |
| --- | --- | --- | --- | --- | --- |
| I_U_ | I_D_ | I_U_ | I_D_ | I_U_ | I_D_ |
| 2.19+06  ±7.35E+04 | 3.92E+06  ±7.35E+04 | 2.88E+06  ±7.35E+04 | 3.42E+06  ±7.35E+04 | 1.33E+06  ±7.35E+04 | 3.70E+06  ±7.35E+04 |

**BiPT229GV461F AMPylated (ATP-bound):** p_D_=89±3.4%

| P1 | | P2 | | P3 | |
| --- | --- | --- | --- | --- | --- |
| I_U_ | I_D_ | I_U_ | I_D_ | I_U_ | I_D_ |
| 4.32E+05  ±5.48+04 | 6.08E+06  ±5.48+04 | 7.76E+05  ±5.48+04 | 4.48E+06  ±5.48+04 | 6.28E+05  ±5.48+04 | 4.59E+06  ±5.48+04 |

**BiPT229GV461F AMPylated (ADP-bound):** p_D_=87±3.6%

| P1 | | P2 | | P3 | |
| --- | --- | --- | --- | --- | --- |
| I_U_ | I_D_ | I_U_ | I_D_ | I_U_ | I_D_ |
| 6.44E+05  ±5.50E+04 | 5.62E+06  ±5.50E+04 | 1.11E+06  ±5.50E+04 | 4.86E+06  ±5.50E+04 | 6.47E+05  ±5.50E+04 | 4.87E+06  ±5.50E+04 |
