# Supplementary material for: Allosteric fine-tuning of the conformational equilibrium poises the chaperone BiP for post-translational regulation
Source: eLife. 2017 Oct 24;6:e29430. doi: 10.7554/eLife.29430 (PMC5655141; doi:10.7554/eLife.29430)
Supplement: Figure 5—source data 2. — (Top) Thermodynamic parameters of ATP and ADP binding obtained from ITC experiments for FL BiP* and its non-AMPylated and AMPylated V461F variant; the measurements were repeated three times and the standard deviations for all parameters were less than 10%. (Bottom) Ratio of populations of the domain-docked (pD) and undocked (pU) conformations obtained from the analysis of NMR peak intensities (Figure 4—source data 1 and Figure 5—source data 1). (Grey) The experimental free energy of nucleotide binding and domain docking plotted in Figure 5B. The free energy of binding was calculated as ΔG(binding)/RT= Ln(Kd), where Kd is ADP or ATP binding constant obtained by ITC, R is the ideal gas constant, and T is the temperature in K; the free energy of domain docking was calculated from populations of the domain-undocked and -docked conformations obtained from the NMR analysis using the following equation: ΔG(docking)/RT= −Ln(pD/pu). [file elife-29430-fig5-data2.docx]

**Figure 5–source data 2**

**Analysis of the thermodynamic linkage between domain docking and nucleotide binding.**

(Top) Thermodynamic parameters of ATP and ADP binding obtained from ITC experiments for FL BiP* and its non-AMPylated and AMPylated V461F variant; the measurements were repeated three times and the standard deviations for all parameters were less than 10%.

(Bottom) Ratio of populations of the domain-docked (p_D_) and undocked (p_U_) conformations obtained from the analysis of NMR peak intensities (Figure 4–table supplement 6 and Figure 5–table supplement 3).

(Grey) The experimental free energy of nucleotide binding and domain docking plotted in Figure 5B. The free energy of binding was calculated as $\Delta G(binding)/RT= Ln(K_{d})$, where K_d_ is ADP or ATP binding constant obtained by ITC, R is the ideal gas constant, and T is the temperature in K; the free energy of domain docking was calculated from populations of the domain-undocked and -docked conformations obtained from the NMR analysis using the following equation: $\Delta G(docking)/RT= -Ln(p_{D}/p_{u})$.

|  | **ANP** | **K_d_ (µM)** | **∆G (kcal/mol)** | **∆H (kcal/mol)** | **-T∆S (kcal/mol)** |
| --- | --- | --- | --- | --- | --- |
| **BiP* FL** | ATP | 0.80 | -8.3 | 11.4 | -19.7 |
|  | ADP | 5.73 | -7.1 | -7.4 | 0.3 |
| **BiP*V461F** | ATP | 0.21 | -9.1 | 7.0 | -16.1 |
|  | ADP | 4.49 | -7.3 | -5.9 | 1.4 |
| **BiP*V461F**  **AMPylated** | ATP | 0.07 | -9.8 | 6.7 | -16.5 |
|  | ADP | 2.36 | -7.7 | -6.9 | -0.8 |
|  |  | **p_D_/p_U_** | **∆G/RT(docking)** | **∆G/RT (binding)** |  |
| **BiP* FL** | ATP | 1.13 | 0.12 | -14.04 |  |
|  | ADP | 0.23 | -1.47 | -12.07 |  |
| **BiP*V461F** | ATP | 1.94 | 0.66 | -15.38 |  |
|  | ADP | 0.96 | -0.04 | -12.31 |  |
| **BiP*V461F**  **AMPylated** | ATP | 8.09 | 2.09 | -16.47 |  |
|  | ADP | 6.69 | 1.90 | -12.96 |  |
